# Corrosion Behavior and Susceptibility to Stress Corrosion Cracking of Leaded and Lead-Free Brasses in Simulated Drinking Water

**DOI:** 10.3390/ma15010144

**Published:** 2021-12-25

**Authors:** Jamal Choucri, Andrea Balbo, Federica Zanotto, Vincenzo Grassi, Mohamed Ebn Touhami, Ilyass Mansouri, Cecilia Monticelli

**Affiliations:** 1 Corrosion and Metallurgy Study Centre “A. Daccò”, University of Ferrara, 44121 Ferrara, Italy; j.choucri@libero.it (J.C.); andrea.balbo@unife.it (A.B.); zntfrc@unife.it (F.Z.); vincenzo.grassi@unife.it (V.G.); 2 Laboratory of Materials Engineering and Environment, Modelling and Application, University Ibn Tofail, Kenitra 14000, Morocco; mohamed.ebntouhami@uit.ac.ma; 3 International Institute for Water and Sanitation (IEA), National Office of Electricity and Potable Water, Rabat 10220, Morocco; xilyas72@gmail.com

**Keywords:** brass, corrosion, simulated drinking water, chloride, stress corrosion cracking, electrochemical impedance spectroscopy, slow strain rate test

## Abstract

Duplex α + β’ brasses are widely used in drinking water distribution systems for tube fittings, valves, and ancillaries because they are low cost, easy to fabricate, and exhibit high mechanical strength. However, depending on application conditions and alloy composition, they may undergo dealloying and stress corrosion cracking. In this research, three different brass types, two leaded (CW617N and CW602N) alloys and one lead-free brass (CW724R), were investigated to assess their corrosion behavior and susceptibility to stress corrosion cracking (SCC) in simulated drinking water (SDW) solutions containing different chloride concentrations, compatible with drinking water composition requirements according to Moroccan standard NM 03.7.001. The corrosion behavior was assessed by electrochemical tests such as polarization curve recording and electrochemical impedance spectroscopy (EIS) monitoring, coupled to SEM-EDS surface observations. The susceptibility to SCC was investigated by slow strain rate tests (SSRT). The tests showed that corrosion was mainly under diffusion control and chlorides slightly accelerated corrosion rates. All alloys, and particularly CW617N, were affected by SCC under the testing conditions adopted and in general the SCC susceptibility increased at increasing chloride concentration.

## 1. Introduction

Duplex α + β’ brasses are widely used in drinking water distribution systems for tube fittings, parts of pumps, valves, faucets, and ancillaries because they are low cost, easy to fabricate, and exhibit high mechanical strength. However, depending on their composition, they may undergo different corrosion forms, such as dealloying and stress corrosion cracking and may determine human health concerns due to lead and, in the case of traditional dezincification resistant (DZR) alloys, arsenic release in drinking water. New stricter regulations introduced in some areas of the world pushed the production of new low-lead or lead-free DZR alloys.

Concerning dezincification of α and α + β’ brasses, two mechanisms were proposed, that is, selective Zn dissolution [1,2,3] and a more widely accepted selective dissolution model consisting in the initial dissolution of both Zn and Cu, followed by Cu redeposition [4,5,6,7,8]. According to some authors [9,10], the two mechanisms of dezincification are actually both operative on α, α + β’, and β’ brasses under certain conditions of potential and pH.

Silicon brasses with various compositions were developed to induce grain refining and strength increase [11] or to produce non-toxic Pb- and As-free alloys with good machinability [12] and dezincification resistance [8]. CuZn21Si3P is a dezincification resistant brass with α + κ microstructure, where κ is a hard Si-rich phase. Its resistance to selective Zn leaching is ensured by the “phosphorus cycle” adopted as an alternative to the analogous “arsenic cycle” [13]. Actually, in this alloy a significant dealloying process cannot be avoided during long immersions (150 days) in simulated drinking water (SDW) [14].

Under the concomitant presence of stress and aggressive environment, brass dezincification is often reported to accompany and interact with stress corrosion cracking (SCC). In particular, dezincification associated to SCC was documented in ammonia solution [15,16,17,18], drinking water [14,19], and in solutions of chlorides [20], fluorides [21], perchlorates [20], and molybdates [22].

The interaction between these two corrosion forms was attributed to different phenomena, such as (1) the dealloying-induced additive tensile stress at the metal/dezincified layer interface and (2) the enhanced surface diffusivity. The additive tensile stress arises because of the enhanced formation of vacancies during the selective Zn dissolution and causes rupture of the brittle dezincified layer [16,20,23,24,25] or crack initiation due to easier dislocation motion [17,21,26]. The enhanced surface atom mobility is ascribed to the selective Zn dissolution and enhanced surface concentration of vacancies, which can be captured at the crack tip, thus inducing crack growth [27,28]. According to some authors [15], dealloying and SCC stimulate each other synergistically: strained regions are more prone to selective Zn dissolution, while the obtained brittle Zn-depleted Cu film favors crack initiation and propagation.

Dezincification is not always present during SCC, suggesting that other SCC mechanisms can operate independently of the presence or absence of dezincification. The mechanochemical mechanism involves the fracture of the passive film under the action of tensile stress, followed by metal dissolution at the film fracture site, leading to crack initiation and propagation [21]. The adsorption mechanism first hypothesizes the adsorption of chemical species at the crack tip, then the subsequent weakening of local interatomic bonding, and finally the initiation and propagation of brittle fracture at reduced stress levels [29].

In this research, three different brass types, two leaded (CW617N and CW602N, with composition CuZn40Pb2 and CuZn36Pb2As, respectively) alloys and one lead-free brass (CW724R, with composition CuZn21Si3P), were investigated to assess their corrosion behavior and susceptibility to SCC in simulated drinking water (SDW) solutions with different chloride contents ranging from 100 to 700 ppm. Previous tests carried out on CW602N and CW724R in 400 ppm chloride SDW detected general corrosion and extensive dealloying. After 150 day immersions, they resulted covered by a surface film which according to SEM-EDS analyses contained oxides and chlorides. Surface Si-enrichment likely connected to silicate deposition was also detected on CW724R [14].

## 2. Materials and Methods

### 2.1. Materials and Aggressive Environments

**Figure 1:**
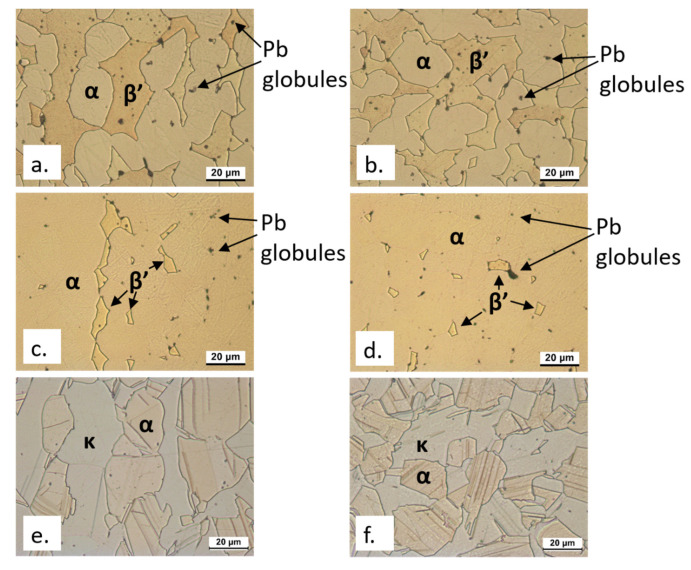
OM microstructures of etched longitudinal (**a**,**c**,**e**) and transverse (**b**,**d**,**f**) sections of CW617N (CuZn40Pb2: (**a**,**b**)), CW602N (CuZn36Pb2As: (**c**,**d**)), and CW724R (CuZn21Si3P: (**e**,**f**)) samples. The longitudinal direction in (**a**,**c**,**e**) is the vertical one.

The composition of the investigated alloys, as evaluated by glow discharge optical emission spectrometry (GDOES), is shown in Table 1, while Figure 1 exhibits the alloy microstructures both in transverse (T) and longitudinal (L) sections.

The alloy CW617N and the dezincification-resistant CW602N evidenced α + β’ microstructures (that is mixed face-centered cubic, fcc, and body-centered cubic, bcc) with dispersed Pb globules, but the amounts of β’ were quite different, that is 62 ± 3% in the former and 10 ± 2% in the latter alloy (as calculated by image analysis software). CW724R exhibited about 40 ± 2% κ phase (grey in color in Figure 1) and balance α phase (pale pink in Figure 1). κ is a hard Si-rich phase, with close-packed hexagonal (cph) lattice and atomic composition close to the stoichiometry Cu8Zn2Si [8]. In CW724R, SEM-EDS analysis also detected small particles of a third bright phase, located at the grain boundaries, corresponding to the cubic γ phase with nominal composition Cu4ZnSi [14].

Two types of specimens were prepared starting from rods of 35 mm diameter.

For electrochemical tests, electrodes with longitudinal surface orientation were obtained by soldering a copper wire onto the back of parallelepiped brass samples with a square basis of 1 cm^2^ (exposed surface). These samples were embedded in epoxy resin and ground by SiC emery paper and finally they were polished with 1 μm diamond powder to reach a mirror finish. In these tests, the solution volume was 250 cm^3^ and no stirring was applied.

**Figure 2:**
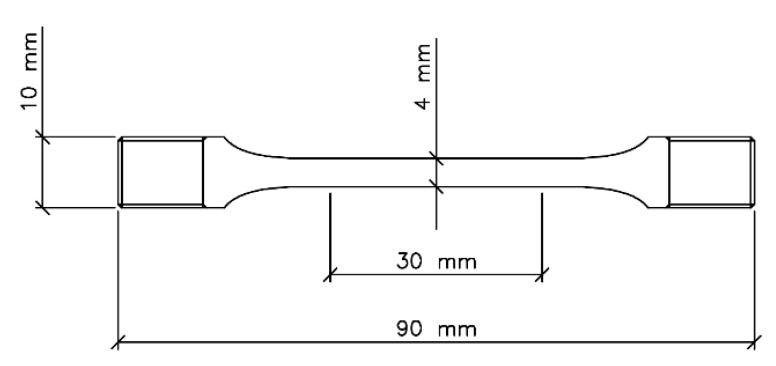
Scheme of the tensile specimens adopted in slow strain rate tests (SSRT).

For slow strain rate tests (SSRT), tensile specimens were prepared with an overall length of 90 mm and diameter of the grip sections of 10 mm, while the gauge portion had length and diameter of 30 mm and 4 mm, respectively (Figure 2). The gauge portion of the tensile specimens was ground parallel to the tensile direction down to 1200 grit emery paper, degreased with acetone, and covered by a two-component epoxy varnish leaving an exposed central region of about 3 cm^2^. In these tests, the solution volume was 500 cm^3^ and again no stirring was applied.

The compositions of the aggressive solutions were:

- 100 ppm Cl^−^ (0.0028 M NaCl) + 400 ppm SO_4_^2−^ (0.0042 M Na_2_SO_4_) + 50 ppm NO_3_^−^ (0.00081 M NaNO_3_);
- 400 ppm Cl^−^ (0.0112 M NaCl) + 400 ppm SO_4_^2−^ (0.0042 M Na_2_SO_4_) + 50 ppm NO_3_^−^ (0.00081 M NaNO_3_);
- 700 ppm Cl^−^ (0.0196 M NaCl) + 400 ppm SO_4_^2−^ (0.0042 M Na_2_SO_4_) + 50 ppm NO_3_^−^ (0.00081 M NaNO_3_).

The anion concentrations were within the accepted ranges for drinking water requirements, according to Moroccan standard NM 03.7.001. The solution pH was about 6.5. All the tests were carried out at 25 °C.

### 2.2. SEM-EDS Surface Analyses

Unpolarized electrodes exposed for 24 h to the aggressive solutions were examined by scanning electron microscope (SEM) (Zeiss EVO MA15, Zeiss, Oberkochen, Germany), equipped by an energy dispersive X-ray spectroscopy (EDS) system (Oxford Aztec, Oxford, UK) to investigate the morphology of the corrosion attack and the nature of the surface corrosion products.

### 2.3. Electrochemical Tests

The electrochemical tests were carried out in a conventional thermostated three-electrode cell by using a PARSTAT 2273 potentiostat/galvanostat (Ametek, Berwyn, PA, USA), piloted by PowerSuite software (version 2.58, Advanced Measurement Technology, Inc., Oak Ridge, TN, USA). The reference and auxiliary electrodes were a saturated calomel electrode (SCE) and a Pt sheet, respectively. All the potential values quoted in the text are referred to the SCE.

Cathodic and anodic polarization curves were recorded at a scan rate of 0.167 mV/s on separated electrodes, always starting from the corrosion potential (*E_cor_*), after 1 and 24 h of immersion in SDW solutions. The Levenburg–Marquardt (LEV) method was applied (by software SAI CView v.3.5c, Scribner Associates Inc., Southern Pines, NC, USA) to properly fit the coupled anodic and cathodic polarization curves to the following equation:

(1) $$i=i_{cor}\;\left(10^{\frac{E_{cor}-E}{\beta_{a}}}-10^{\frac{E_{cor}-E}{\beta_{c}}}\right)$$

in order to evaluate the corrosion current density, *i_cor_* and the anodic and cathodic Tafel slopes (*β_a_* and *β_c_*) [30].

Electrochemical impedance spectroscopy (EIS) tests were performed after 1, 4, 12, and 24 h of immersion. The tests were carried out at *E_cor_* by adopting a voltage perturbation amplitude of 10 mV (rms), in the frequency range 10^4^–10^−2^ Hz (1 h, 4 h, and 12 h immersion) or 10^4^–10^−3^ Hz (24 h immersion), with ten points per frequency decade. EIS spectra were fitted by SAI ZView v.3.5c software (Scribner Associates Inc.) according to the most suitable equivalent circuit, as described in the text.

At least two experiments were carried out under each experimental condition.

### 2.4. Slow Strain Rate Tests (SSRT)

Slow strain rate tests (SSRT) were performed by inserting the specimens in a thermostated cell, either not containing (reference tests in air) or containing the aggressive solutions. During the tests in solutions, *E_cor_* was continuously monitored.

The susceptibility to SCC of these brasses was studied at a strain rate of 0.75 × 10^−6^ s^−1^. It was quantified by the R ratio between the percentage elongation to fracture (ε_f_%) in the test solution (ε_fs_%) and that in air (ε_fa_%).

After all SSRT, the fracture surface and the longitudinal sections of the specimen gauges were examined by SEM and optical microscope (OM, Leica Microsystems GmbH, Wetzlar, Germany), respectively.

## 3. Results

### 3.1. SEM-EDS Surface Analyses

**Figure 3:**
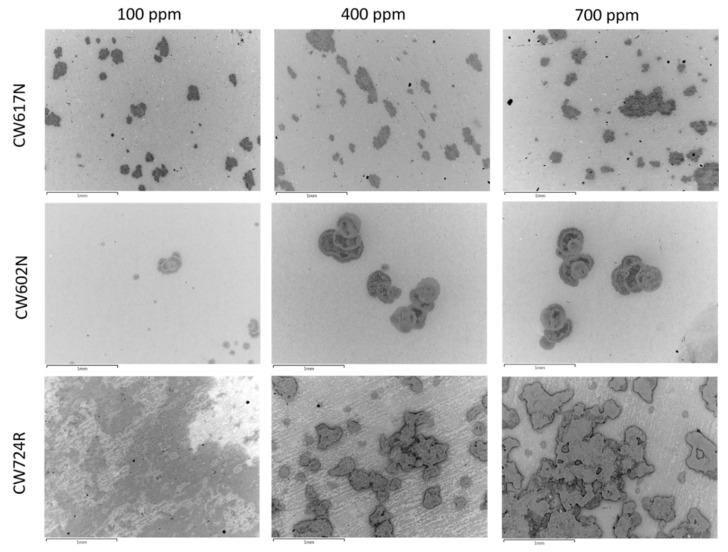
BS-SEM images of brass specimens after 24 h immersions in SDW solutions containing different chloride concentrations.

The backscattered electron (BSE) SEM images of the specimens immersed for 24 h in SDW solutions containing 100 ppm, 400 ppm, and 700 ppm chlorides are collected in Figure 3.

**Figure 4:**
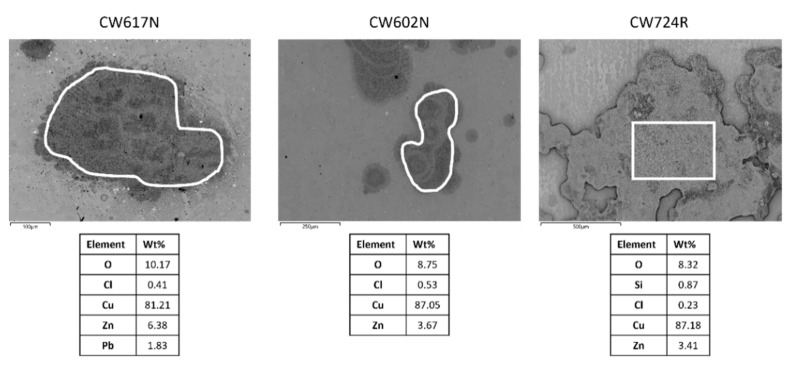
SEM-EDS analyses carried out in correspondence of dark spots formed on brasses after 24 h immersions in SDW solutions at 700 ppm chlorides.

The Figure clearly shows the onset of dealligation spots under all exposure conditions and particularly at high chloride concentrations. This is confirmed in Figure 4 which reports, as an example, the SEM-EDS analyses carried out in correspondence of the dark spots obtained at 700 ppm chlorides. In these regions, a strong zinc (for all alloys) and silicon (in the case of CW724R) depletion is detected.

SEM-EDS surface analyses also revealed preferential attacks on lead globules, particularly at high chloride concentrations, and the overall growth of surface oxide films. In addition to the alloying elements and oxygen, the surface presence of chlorine was also detected (usually within 0.5–0.6 wt%) in correspondence of dealloyed regions, suggesting the adsorption of chlorides or chloride salt deposition. No sulphur or nitrogen were detected indicating the absence of sulfates and nitrates.

### 3.2. Polarization Curves

**Figure 5:**
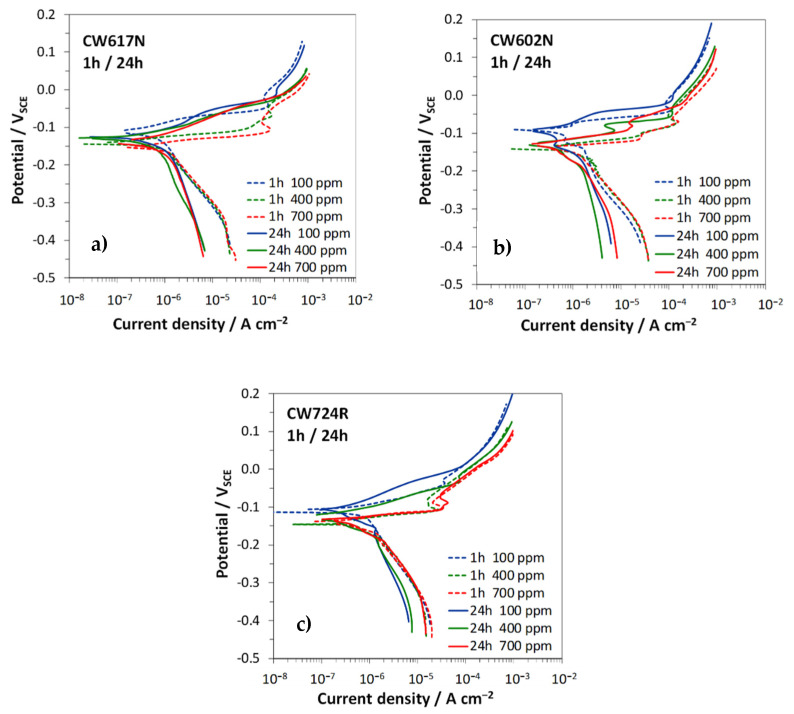
Polarization curves recorded on CW617N (**a**), CW602N (**b**), and CW724R (**c**) after 1 h (dashed lines) and 24 h (solid lines) immersion in the SDW solutions, at the three different chloride concentrations.

Figure 5 collects the polarization curves recorded after 1 h and 24 h in SDW with different chloride contents on the investigated brass alloys, while Table 2 collects *E_cor_* and corrosion currents (*i_cor_*) values, together with the anodic (*β_a_*) and cathodic (*β_c_*) Tafel slopes.

In general, the alloys exhibited a similar dependence of the curve shapes from time and chloride concentration. In particular, at 1 h immersion (Figure 5, dashed lines and Table 2), the anodic slopes were low, particularly in solutions with 400 and 700 ppm chlorides and markedly increasing anodic currents were recorded at increasing chloride concentrations. The cathodic polarization curves, connected to the oxygen reduction reaction, were scarcely affected by the chloride concentrations. At these short immersion times, they reached the limiting current of oxygen reduction only at high cathodic overvoltages (at about −0.35 V), where the absolute value of the slopes tended to infinity.

This suggests that at E_cor_ oxygen reduction was mainly under activation control.

After 24 h immersion, the anodic and cathodic currents (Figure 5, solid lines) and, consequently, the i_cor_ values (Table 2) were slightly lower than those at short immersion time and the cathodic reaction appeared to be mainly under diffusion control, as suggested by the increase in the cathodic slopes.

Concerning the influence of chlorides, Table 2 evidences that the increase in chloride concentration determined a small but evident i_cor_ stimulation on all tested alloys.

### 3.3. Electrochemical Impedance Spectroscopy Tests

**Figure 6:**
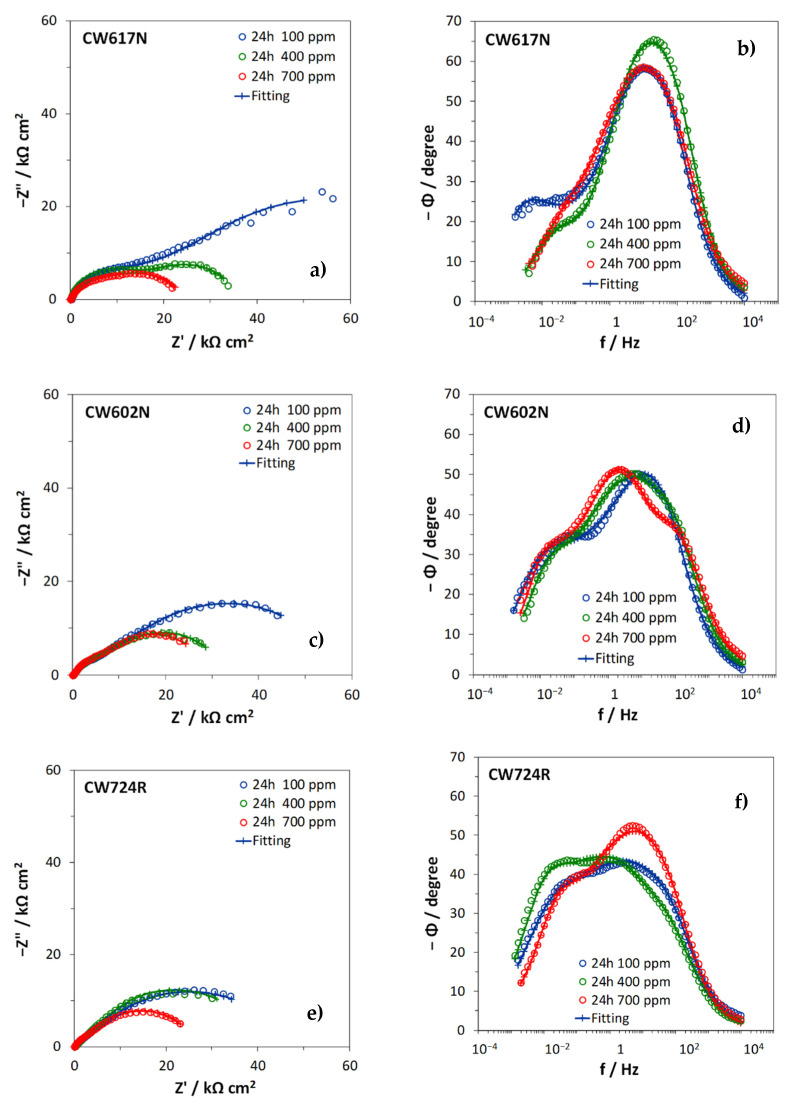
EIS spectra recorded on CW617N (**a**,**b**), CW602N (**c**,**d**), and CW724R (**e**,**f**) after 24 h immersion in SDW solutions, at the three different chloride concentrations.

Figure 6 collects the Nyquist and Bode (phase angle) plots of the impedance spectra recorded after 24 h immersion in the different SDW solutions.

The spectra recorded on CW617N at all chloride concentrations and on CW602N at 100 ppm chlorides clearly exhibited two arcs in the Nyquist plots and, in accordance, two peaks in the Bode plots of the phase angle, centered respectively at frequencies of 0.01 Hz or lower (low frequency, LF, time constant) and 1–30 Hz (medium frequency, MF, time constant). One more time constant centered at about 100–150 Hz (high frequency, HF, time constant) occurred in the other spectra (although not always very evident) and was particularly evident on CW602N at 700 ppm.

**Figure 7:**
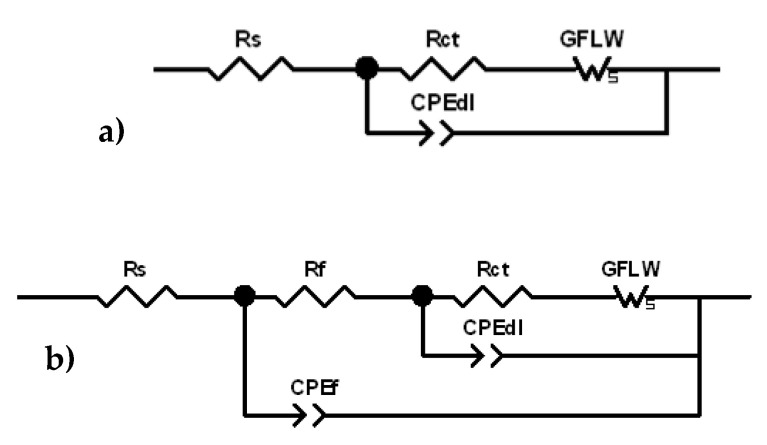
Equivalent circuits (EC) used to fit the electrochemical impedance spectroscopy (EIS) spectra: (**a**) two-time-constant EC; (**b**) three-time-constant EC.

In a previous research paper [14], similar results were obtained on CW602N and CW724R during 150 days of immersion in 400 ppm chloride SDW solutions. In that case, it was shown that the HF time constant shifted to 10^3^ or 10^4^ Hz at long immersion times particularly on silicon brass. On the basis of this previous paper and other literature information [31,32,33,34,35], the spectra were fitted by the equivalent circuits (EC) reported in Figure 7a (two time constants) and Figure 7b (three time constants). In these EC, R_s_ is the solution resistance, the R_f_−CPE_f_ parallel couple describes the dielectric properties of the surface corrosion product film (HF time constant), the R_ct_–CPE_dl_ parallel couple provides information about the charge transfer process at the metal/electrolyte interface at the bottom of the film pores (MF time constant), and GFLW is a generalized finite length Warburg element, introduced in series to the charge transfer resistance, R_ct_, to fit the LF time constant and attributed to diffusion.

The impedance of the *CPE* element is defined as:

(2) $$Z_{CPE}=Y^{-1}{\left(j\omega \right)}^{-n}$$

where *ω* = 2πf is the angular frequency, *j* = √ (−1) is the imaginary unit, *Y* is a frequency independent value, and *n* is a fit parameter with values in the range 0 ≤ *n* ≤ 1, which measures the element deviation from the ideal capacitive behavior (exhibiting *n* = 1).

It is introduced instead of pure capacitances to take into account non idealities of the corroding electrode surfaces attributed to insufficient polishing, grain boundaries, and surface impurities [36]. The associated capacitances can be calculated by the general equation [37]:

(3) $$C={\left(R^{1-n}\;Y\right)}^{\frac{1}{n}}$$

where Y and n are the parameters of the CPE element and R is the resistance involved in the considered R-CPE couple. The GFLW element is generally used to take into account diffusion processes over paths of finite length and its impedance is expressed by the following analytical expression:

(4) $$Z_{GFLW}=R_{W}\frac{tanh{\left(j\omega T\right)}^{p}}{{\left(j\omega T\right)}^{p}}$$

where T is a time constant, $R_{W}$ is a resistance, and *p* is an exponent which can vary in the range 0 < *p* < 1. For *p* = 0.5, T = L^2^/D, where L is the thickness of the diffusion layer and D is the diffusion coefficient [38].

**Figure 8:**
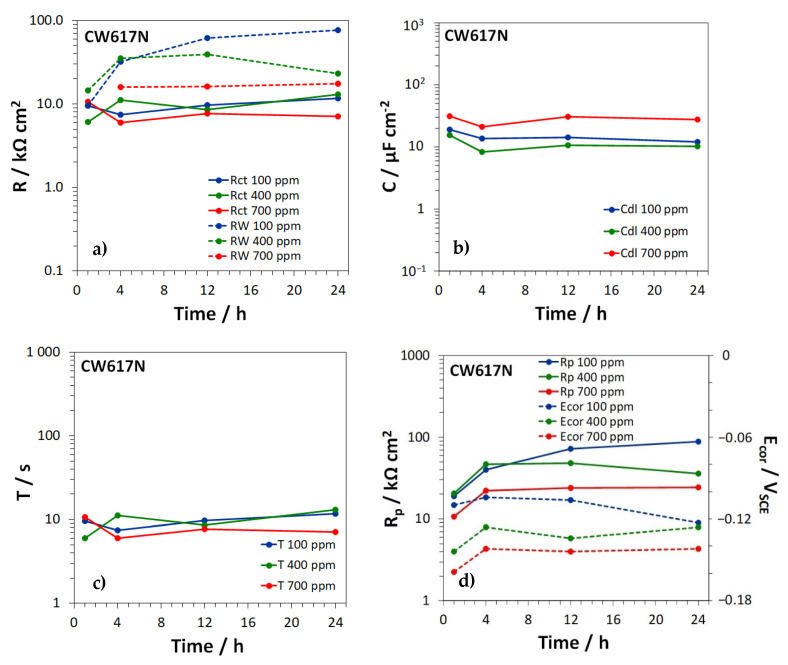
Time dependence of (**a**) the charge transfer and diffusion resistances (*R_ct_* and *R_W_*); (**b**) the double layer capacitance (*C_dl_*); (**c**) the *T* parameter of the GFLW element; and (**d**) the polarization resistance (*R_p_*) and corrosion potential values (E_cor_), measured on CW617N during the immersion in SDW with three different chloride concentrations.

**Figure 9:**
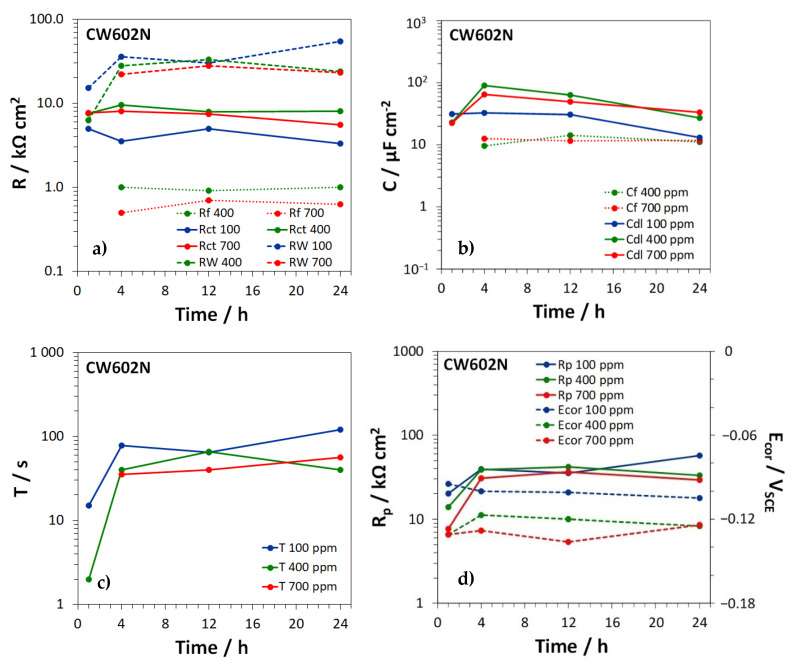
Time dependence of (**a**) the film, charge transfer, and diffusion resistances, (*R_f_*, *R_ct_*, and *R_w_*); (**b**) the film and double layer capacitances (*C_f_* and *C_dl_*); (**c**) the *T* parameter of the GFLW element; and (**d**) the polarization resistances (*R_p_*) and the corrosion potentials (E_cor_), measured on CW 602N during the immersion in SDW with three different chloride concentrations.

**Figure 10:**
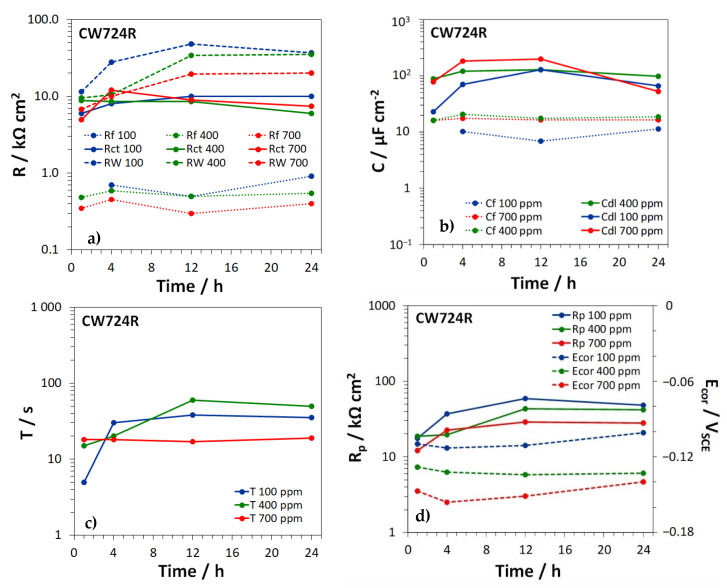
Time dependence of (**a**) the film, charge transfer, and diffusion resistances (*R_f_*, *R_ct_*, and *R_W_*); (**b**) the film and double layer capacitances (*C_f_* and *C_dl_*); (**c**) the *T* parameter of the GFLW element; and (**d**) the polarization resistance (*R_p_*) and corrosion potential values (E_cor_), measured on CW724R during the immersion in SDW with three different chloride concentrations.

For the different brass types, Figure 8, Figure 9 and Figure 10 show the time dependence of the film, charge transfer, and diffusion resistances ($R_{f}$, $R_{ct}$, and $R_{W}$, Figure 8a, Figure 9a and Figure 10a), the film and double layer capacitances (*C_f_* and *C_dl_*, Figure 8b, Figure 9b and Figure 10b) and the *T* parameter of the GFLW element (Figure 8c, Figure 9c and Figure 10c), during the immersion in SDW at different chloride concentrations. *n_f_* values associated to CPE_f_ were in the range 0.80–0.88, while *n_dl_* values, related to CPE_dl_, were somewhat lower (in the range 0.60–0.86). The *p* parameter of the GFLW element remained in the range 0.32–0.50. Polarization resistance ($R_{p}$) values, inversely proportional to the corrosion rates, were calculated as the sum:

$$R_{p}=R_{f}+R_{ct}+R_{W}$$

and are collected in Figure 8d, Figure 9d and Figure 10d for the different alloys, together with the corresponding E_cor_ values.

According to the EIS spectra, no film formed on CW617N (Figure 8a), even if SEM-EDS analyses detected the presence of a surface oxide film on all brass types at all chloride concentrations. This discrepancy was likely due to the relatively high conductivity and thickness of the film on CW617N, which make the film characteristic frequencies higher than those accessible by EIS tests [39]. On the other brasses, oxide films with more or less constant $R_{f}$ values of about 1 KΩ cm^2^ or lower were detected (Figure 9a and Figure 10a). The corresponding *C_f_* values were around 10–12 µF cm^−2^ (Figure 9b and Figure 10b), in good agreement with literature information under similar conditions [14,31].

Even $R_{ct}$ values remained rather constant within the observed time interval (around 10 KΩ cm^2^ or slightly lower) and resulted scarcely affected by the chloride concentrations (Figure 8a, Figure 9a and Figure 10a). The corresponding *C_dl_* values (Figure 8b, Figure 9b and Figure 10b) assumed values of 10–100 µF cm^−2^, again similar to previously achieved ones [14,31]. They were scarcely dependent upon time and often assumed the highest values at the highest chloride concentration, likely due to a more significant chloride adsorption at the metal/solution interface at the bottom of film pores [39].

On all brass types, *R_W_* values were not only much higher than *R_f_*, but also exceeded $R_{ct}$ values, particularly at immersion times longer than 1–4 h (Figure 8a, Figure 9a and Figure 10a). The figures clearly show that *R_W_* values tended to decrease at increasing chloride concentrations, especially on CW617N and CW724R and, in accordance, the lowest *T* values were generally obtained at the highest chloride concentration. This suggests that diffusion processes control brass corrosion rates, mainly at low chloride concentration and long immersion times.

Figure 8d, Figure 9d and Figure 10d clearly indicate that the $R_{p}$ values, which measure the brass corrosion resistance, increased with time and by moving to lower chloride concentrations. These Figures also evidence that the corresponding E_cor_ values always remained in the range −0.160 ÷ −0.090 V, with a tendency to ennoblement at decreasing chloride concentration.

### 3.4. Slow Strain Rate Tests 

**Figure 11:**
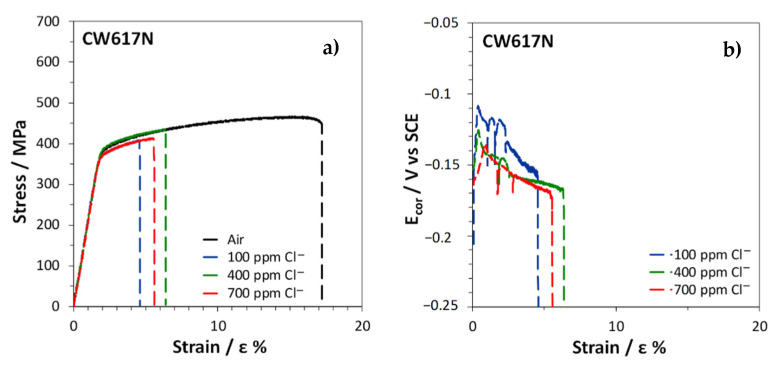
SSRT on CW617N: (**a**) stress vs. strain curves obtained in air and in SDW solutions and (**b**) E_cor_ values recorded in SDW solutions.

The stress–strain curves obtained by SSRT on CW617N exposed to air or immersed in SDW solutions with different chloride concentrations are reported in Figure 11a.

In air, the fracture strain was about 17% (Table 3), suggesting a scarce alloy ductility, due to the high volume fraction of the brittle β’ phase. In solutions, an even lower ductility was obtained with fracture strains ranging within 4 ÷ 6% (Table 3). The R index, corresponding to the ratio of the fracture strain in solution (ε_fs_) to that in air (ε_fa_) was in the range 0.27 ÷ 0.37 (Table 3), indicating a high susceptibility to SCC in these environments. The lowest R value was obtained at the lowest chloride concentration.

The E_cor_ values measured on CW617N during the SSRT in SDW solutions decreased with increasing strain (Figure 11b), reaching about −0.150 ÷ −0.170 V, depending on the chloride concentration in the test solution. The noblest potential values were obtained at the lowest chloride concentration. The progressive E_cor_ reactivation suggests a faster anodic dissolution at increasing alloy deformation.

**Figure 12:**
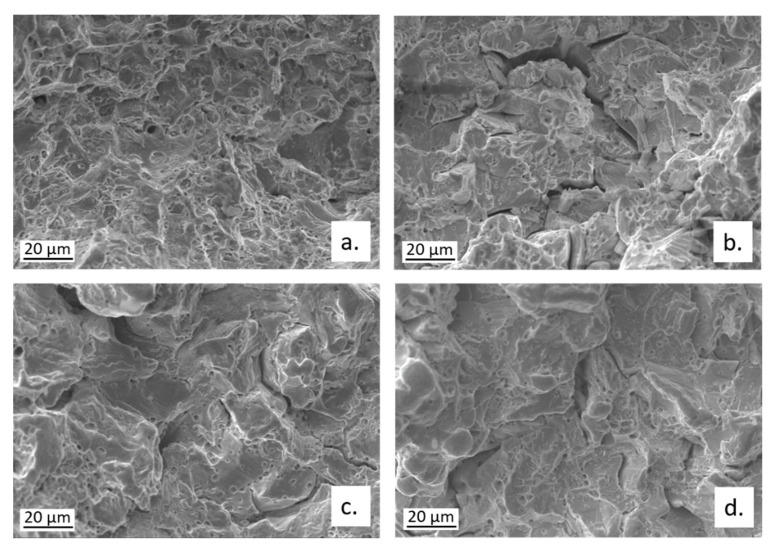
Fractographic analysis of CW617N tensile samples after SSRT carried out in (**a**) air or in SDW solutions containing (**b**) 100 ppm; (**c**) 400 ppm; and (**d**) 700 ppm chlorides.

The fractographic analysis (Figure 12) revealed that the failure occurred through cracks that mainly followed transgranular (TG) paths, even if some intergranular (IG) cracks were also visible.

**Figure 13:**
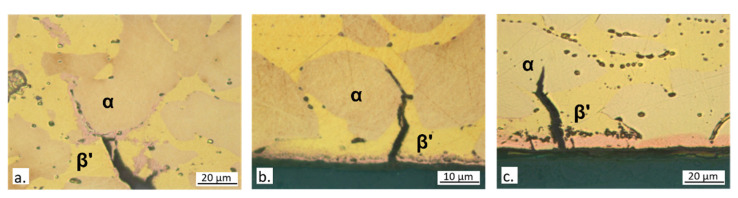
Optical micrographs of sections of CW617N specimens after SSRT in SDW solutions containing (**a**) 100 ppm; (**b**) 400 ppm; and (**c**) 700 ppm chlorides.

Figure 13 shows typical secondary cracks observed in longitudinal sections of specimen gauges. Dezincification was always observed suggesting an interaction between SCC and dezincification. At 400 and particularly at 700 ppm chlorides, large macroscopic surface dezincification spots formed and favored crack formation (Figure 13b,c). At 100 ppm chlorides, where SCC susceptibility was highest (Table 3), surface dezincification was less intense, while it was clearly visible inside the cracks and at the crack tips (Figure 13a), producing a brittle film where the stress intensity was maximum. This could have favored crack propagation. Figure 13b also evidences that in CW617N cracks started on the β’ phase (more easily dezincified), while α caused crack deflection and meandering (Figure 13a).

**Figure 14:**
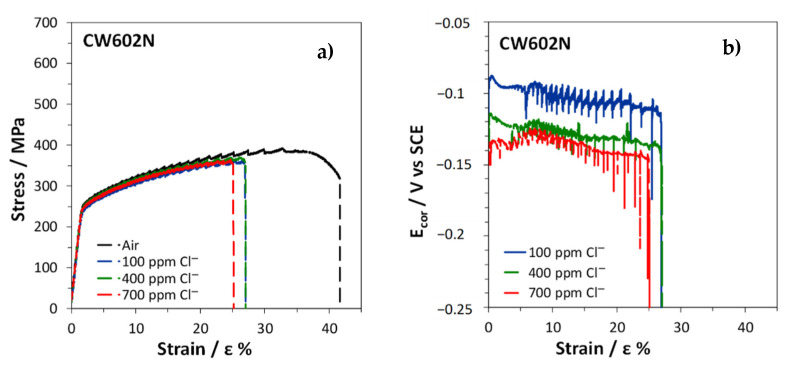
SSRT on CW602N: (**a**) stress vs. strain curves obtained in air and in SDW solutions and (**b**) E_cor_ values recorded in SDW solutions.

Figure 14 shows the stress–strain curves and, in SDW solutions, even the E_cor_ trends obtained on CW602N tensile specimens during the SSRT. This brass was more ductile than CW617N because in air ε_f_% reached 47% (Table 3). The significant reduction of ε_f_% in solutions and the consequently low measured R ratios of 0.60 ÷ 0.65 (Table 3) evidenced that this alloy too is susceptible to SCC, although less than the previous one. The lowest R ratio and therefore the highest SCC susceptibility was achieved in 700 ppm chloride solution.

It is interesting to observe that in both air and solution, jagged stress–strain curves were recorded. This is a mechanical phenomenon known as dynamic strain aging or DSA [40,41,42]. In the case of α and α + β’ brasses it was attributed to the interaction of mobile dislocations with Zn solute atoms. In fact, Zn atoms are slightly larger than Cu atoms and are attracted by the regions around edge dislocations loaded by tensile stresses, allowing a reduction in the overall alloy elastic energy.

When testing is carried out in a particular range of strain rates and temperatures, the solute atoms diffuse quickly enough to trap the moving dislocations dynamically, as deformation occurs. When this happens, the stress must build up again to continue the deformation process, but as a sufficiently high stress is reached dislocation unlocking occurs and the stress drops. In that range of temperature and stress, the dislocations alternately break free from solute atmospheres and are then repinned, producing serrated curves. The absence of DSA in CW617N is likely due to the very high fraction of hard β’ phase (62%) so that freed dislocations cannot move at lower stress levels and stress drops are not observed [43].

**Figure 15:**
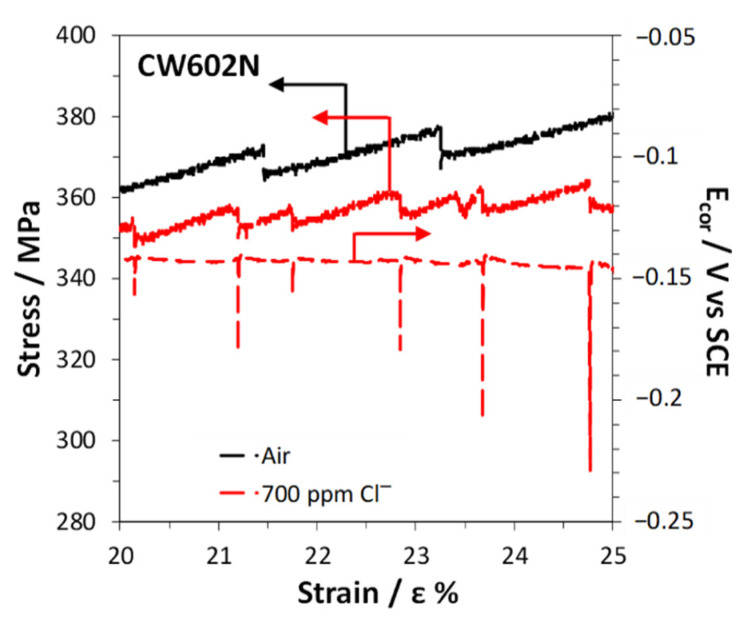
Enlarged view of portions of both the stress vs. strain curves obtained in air and in SDW with 700 ppm chlorides on CW602N and the E_cor_ vs. strain curve of the alloy in the same solution.

In solutions, two observations can be done: (1) the stress drops are concomitant to E_cor_ drops and (2) these drops are more frequent than in air (Figure 15). The first observation suggests that in this alloy mechanical slip determines surface film rupture on the alloy, followed by accelerated dissolution at new active sites and, consequently, E_cor_ reactivation. Then, fast film healing and E_cor_ ennobling occur. The other observation involves a corrosion stimulation of mechanical slip events. This latter phenomenon is not new and was attributed by some authors [20,44] to the formation of pits which can produce stress concentration and can consequently stimulate mechanical slip. Other authors found that dealloying stimulated SCC due to the additive stress produced at the dealloyed brittle film/substrate interface. An additive stress could justify easier mechanical slips [17,21,26], the consequent formation of cracks in the brittle film, and more frequent stress and E_cor_ transients.

**Figure 16:**
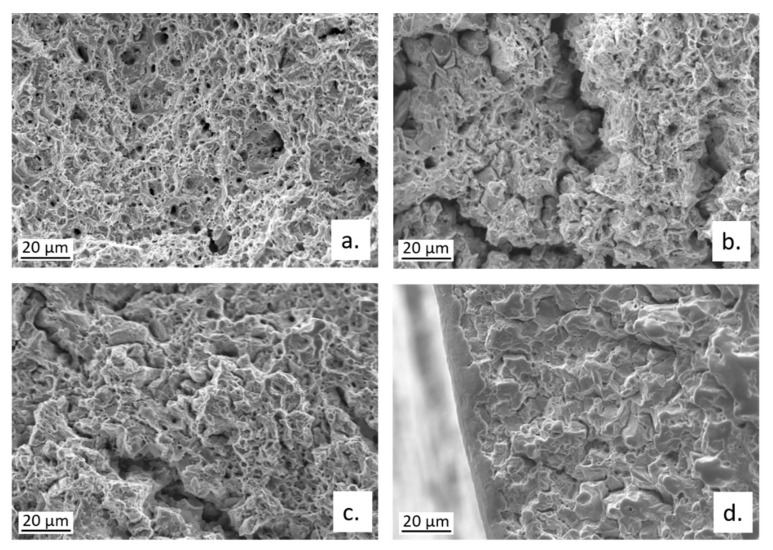
Fractographic analysis of CW602N tensile samples after SSRT carried out in (**a**) air or in SDW solutions containing (**b**) 100 ppm; (**c**) 400 ppm; and (**d**) 700 ppm chlorides.

The fractographic analysis on broken CW602N tensile specimens revealed that the fracture mode moved from ductile to mixed ductile/brittle, on changing the environment for SSRT from air to SDW solutions. In fact, a transition from a ductile dimpled fracture, to fracture surfaces with shallower dimples, associated with some cleavage fracture areas and multiple cracks is documented in Figure 16. The cracks occurred along both TG and IG paths, as also evident in Figure 16a, showing an optical micrograph of a secondary crack section, after microstructural etching.

**Figure 17:**
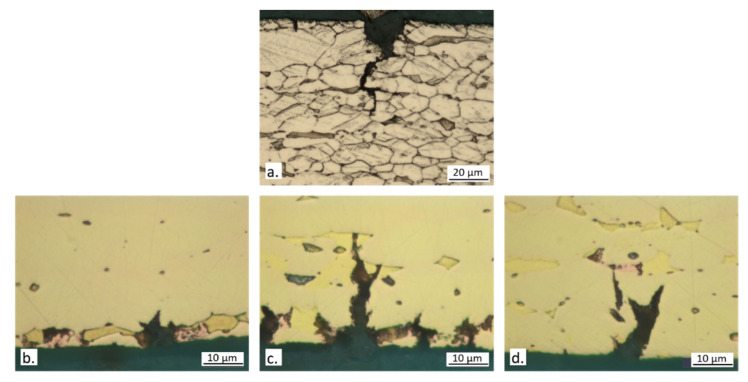
Optical micrographs of secondary cracks on CW602N section surfaces after SSRT in SDW solutions containing (**a**) 400 ppm (surface etching by acid FeCl3 solution to evidence grain boundaries); (**b**) 100 ppm; (**c**) 400 ppm; and (**d**) 700 ppm chlorides.

Figure 17a–d collecting optical microscope pictures of secondary cracks appear to support both hypotheses concerning the stimulation of mechanical slip due to corrosion. In fact, cracks often originated from dezincified regions localized on the emerging β’ phase (Figure 17b,c) and these localized attacks can lead to the formation of craters (Figure 17a) which can produce stress intensification.

It is interesting to observe that when the cracks cross the β’ phase, which is elongated in the longitudinal specimen direction, dezincification and subsequent oxidation of the β’ phase ensue, thus determining crack tip blunting and crack deviation (Figure 17c,d). This delays the fracture to higher strains and explains the lower SCC susceptibility of the alloy, in comparison with CW617N. At increasing chloride concentration, dezincification propagated more deeply into the material, causing higher SCC susceptibility.

**Figure 18:**
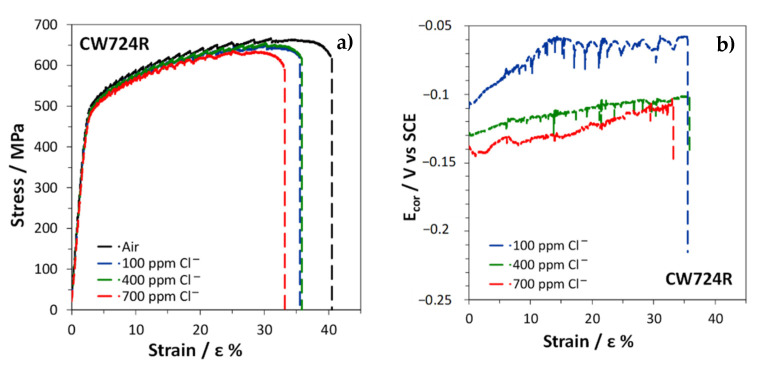
SSRT on CW724R: (**a**) stress vs. strain curves obtained in air and in SDW solutions and (**b**) E_cor_ values recorded in SDW solutions.

Figure 18 collects the stress–strain curves and E_cor_ trends obtained on CW724R. It shows that even on this alloy a serrated plastic flow occurs in both air and solutions, although this alloy, like CW617N, contains a high fraction of hard phase (κ in this case) which should reduce or annul the stress drops. Silicon brass actually contains silicon, besides zinc, as a solute atom. The size of silicon atoms is slightly bigger than that of zinc atoms, so it is reasonable that this element produces stronger interactions with dislocations, with consequent increase in stress drops after dislocation unpinning. This could oppose the effect of the high hard phase fraction. The DSA phenomenon has already been observed in fcc, bcc, as well as in hcp structures [45].

**Figure 19:**
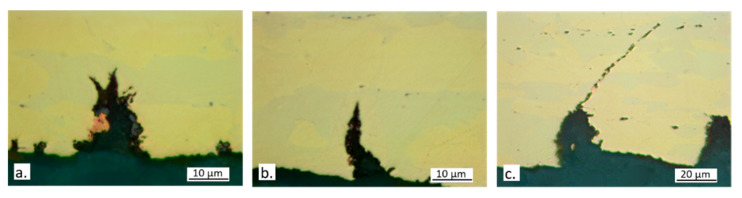
Optical micrographs of sections of CW724R specimens after SSRT in SDW solutions containing (**a**) 100 ppm; (**b**) 400 ppm; and (**c**) 700 ppm chlorides.

The curves in Figure 18a show that CW724R is ductile (ε_f_% = 46 in air) and undergoes limited reductions of ε_f_% in solution, so that high R values of 0.82 ÷ 0.88 were obtained (Table 3). The lowest value was obtained in 700 ppm chloride solution. In spite of these high R ratios, the presence of multiple secondary cracks on the tensile specimens after SSRT (Figure 19) evidenced that even this alloy underwent SCC at all chloride concentrations and particularly at the highest one.

In contrast to the other investigated brasses, E_cor_ values recorded during SSRT (Figure 18b) tended to ennoble at increasing strain. According to some authors [46], this could be due to a noble copper layer redeposited after dezincification. However, it is also possible that on this alloy, where surface silicon tends to form electrically insulating silicates [47], the formation of multiple cracks on the surface film predominantly speeds up the cathodic activity and consequently ennobles E_cor_.

As found on the alloy CW602N, even on CW724R more frequent stress drops were observed in solutions than in air, again suggesting a corrosion stimulation of mechanical slip events. Moreover, simultaneous stress and E_cor_ drops were detected at 100 and 400 ppm, indicating that alternate conditions of mechanical slip and film rupture occurred, followed by accelerated dissolution at new active sites and then repassivation. At higher chloride concentrations, very small E_cor_ oscillations not correlated to stress drops were observed. It is possible that under these conditions the tendency to E_cor_ reactivation after the creation of a new crack is accompanied by a more significant stimulation of the cathodic reaction.

**Figure 20:**
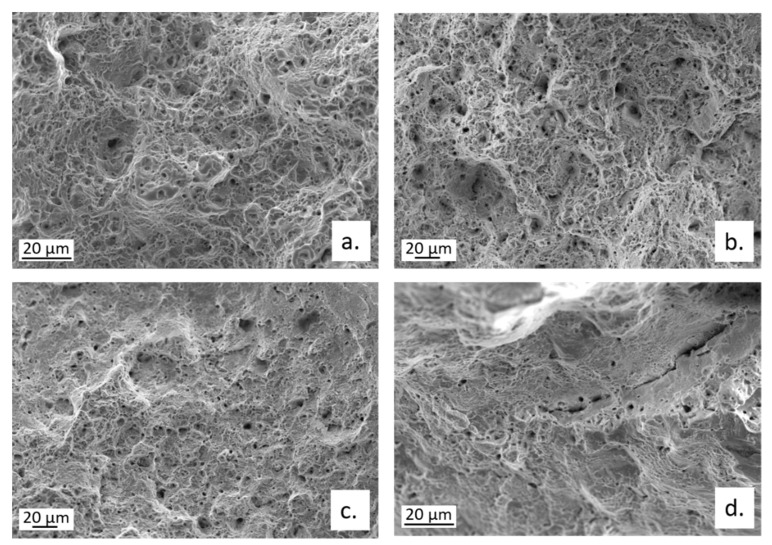
Fractographic analysis of CW724R tensile samples after SSRT carried out in (**a**) air or in SDW solutions containing (**b**) 100 ppm; (**c**) 400 ppm; and (**d**) 700 ppm chlorides.

The fractographic analysis of CW724R specimens revealed the presence of a ductile fracture on the alloy tested in air and a mixed ductile/cleavage fracture in solutions (Figure 20). No evidence of IG cracking was observed.

## 4. Discussion

**Figure 21:**
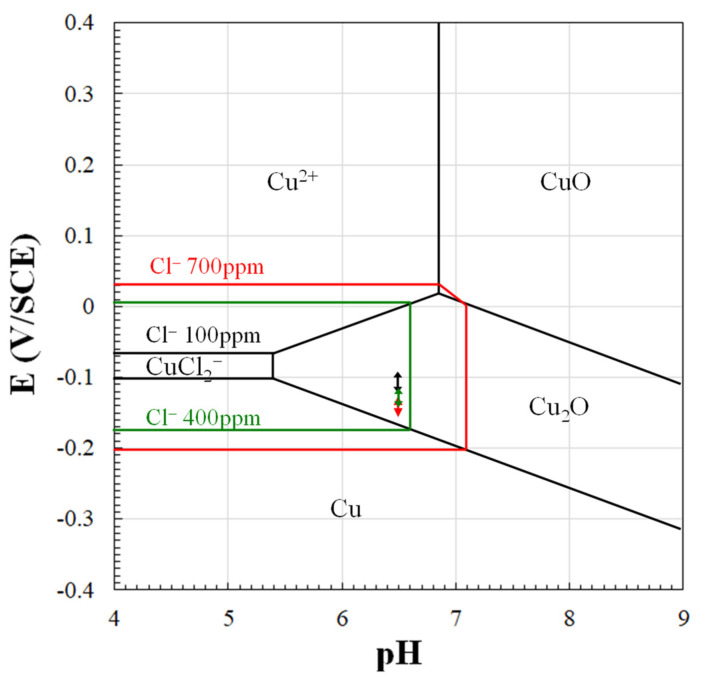
Potential-pH diagram for the system Cu-Cl^−^-H_2_O at 25 °C for the three different chloride concentrations. The black, green, and red lines delimit the CuCl_2_^−^ stability region at 100, 400, and 700 ppm, respectively. The double arrow lines at pH 6.5 show the range of E_cor_ variability for the alloys during the 24 h immersion in 100 (black double arrow line), 400 (green double arrow line), or 700 ppm chlorides (black double arrow line). Figure constructed for a total dissolved Cu species activity of 10^−6^ M.

Figure 21 shows the potential-pH diagram for the system Cu/Cl^−^/H_2_O—at 25 °C at the three chloride concentrations of the SDW solutions. In particular, black, green, and red lines respectively delimit the CuCl_2_^−^ stability region at the chloride concentration of 100 ppm, 400 ppm, and 700 ppm. In the graph, the black, green, and red double arrow lines evidence the variability ranges of E_cor_ for the alloys during the 24 h immersion in SDW solutions (from Figure 8d, Figure 9d and Figure 10d).

The figure evidences that at 100 ppm chlorides on all alloys the formation of a Cu_2_O surface film is predicted, due to the reaction:

2Cu + H_2_O ⇄ Cu_2_O + 2H^+^ + 2 e^−^ (5)

At higher chloride concentrations, copper dissolution is favored by the formation of the CuCl_2_^−^ complex that, according to different authors, can form directly [48,49,50]:

Cu + 2 Cl^−^ ⇄ CuCl_2_^−^ + e^−^ (6)

or via a CuCl_ads_ [51,52,53] intermediate:

Cu + Cl^−^ ⇄ CuCl_ads_ + e^−^ CuCl_ads_ + Cl^−^ ⇄ CuCl_2_^−^ (7)

CuCl_2_^−^ can in turn convert into Cu_2_O due to the surface cathodic alkalinity:

2 CuCl_2_^−^ + 2 OH^−^ ⟶ Cu_2_O + H_2_O + 4 Cl^−^ (8)

This thermodynamic information agrees with the results of electrochemical tests showing decreasing R_p_ values (Figure 8d, Figure 9d and Figure 10d) and higher corrosion rates (Table 2) at increasing chloride concentrations.

Preferential zinc (for CW617N and CW602N) and zinc and silicon (for CW724R) dissolution accompanies copper dissolution due to the thermodynamic activity of these elements under the potential/pH conditions here adopted [54], as confirmed by the dealloying spots evidenced by SEM observations after immersions of only 24 h (Figure 3 and Figure 4).

The analysis of Figure 8a, Figure 9a and Figure 10a shows that brass corrosion rates are under a mixed charge transfer/diffusion control with diffusion being the slowest phenomenon at times longer than 1 h. This latter process may involve mass transport of both oxygen to the electrode surface and CuCl_2_^−^ ions away from the electrode [51].

The SSRT evidence that both CW602N and CW724R underwent DSA in air and SDW solutions, with frequent stress drops and subsequent recoveries to higher stress values. This phenomenon is connected to the dynamic locking and unlocking of dislocations by solute atoms at increasing strain, which determine discontinuous mechanical slips. On CW617N, DSA was not evident likely due to the high fraction of hard β’ phase, which prevented appreciable stress drops. The presence of a serrated plastic flow in both air and solutions on CW724R, in spite of the high fraction of the hard κ phase, is reasonably connected to the presence of silicon as a solute. In fact, silicon has higher atomic dimensions than zinc and likely contrasts the effect of the high hard phase fraction by determining stronger interactions with dislocations, with consequent increase in stress drops after dislocation unpinning.

In solutions, all brasses suffered from SCC in the environments considered and corrosion clearly stimulated mechanical slip events on CW602N (Figure 15) and CW724R. This latter phenomenon can be reasonably interpreted by the dealloying-induced additive tensile stress model, which is compatible with the discontinuity of crack propagation, well evidenced by the presence of concomitant stress and E_cor_ drops (Figure 14, Figure 15 and Figure 18). In fact, the alternative enhanced surface diffusivity model of SCC would involve a continuous crack growth due to dealloying, progressive accumulation of surface vacancies, and vacancy diffusion at the crack tips.

On CW617N, extensive dezincification and significant SCC susceptibility were found, but the discontinuity of the crack growth in SDW solutions was not discernible in the stress/strain and E_cor_/strain curves. Thus, it was not possible to safely identify the dealloying-activated phenomenon favoring SCC.

## 5. Conclusions

The corrosion behavior and SCC susceptibility of two leaded (CW617N and CW602N) alloys and one lead-free silicon brass (CW724R) were investigated in SDW solutions containing different chloride concentrations.

The alloys suffered from spot dealloying that is preferential zinc (alloys CW61N and CW602N) and zinc and silicon (ally CW724R) dissolution already after 24 h immersion. Polarization curves and EIS tests showed that for all brasses corrosion rates slightly increased at increasing chloride concentrations and underwent a limited decrease with immersion time. The kinetics of the corrosion process was mainly under mass transport control.

The SSRT evidenced that all brass types and particularly CW617N exhibited susceptibility to SCC. During the tests, CW602N and CW724R exhibited discontinuous mechanical slips more frequent in SDW solutions than in air, likely due to the onset of dealloying-induced additive tensile stresses.

## Figures and Tables

**Table 1:** UNI-EN specifications and GDOES compositions (as weight percentage) of the studied brasses.

EN Standard Designation	Cu	Zn	Pb	Sn	Fe	Ni	Al	Si	As	P	Other
**CW617N** CuZn40Pb2	58.50	38.90	2.0	0.20	0.30	0.10	0.00	<0.001			<0.10
**CW602N** CuZn36Pb2As	61.70	35.83	1.90	0.11	0.12	0.03	0.02	<0.001	0.09		<0.20
**CW724R** CuZn21Si3P	77.10	19.52	0.00	0.01	0.02	0.00	0.00	3.3	0.00	0.05	<0.10

**Table 2:** Corrosion potentials (E_cor_), corrosion current densities (i_cor_), and anodic (β_a_) and cathodic (β_c_) Tafel slopes obtained from the polarization curves.

Alloy	Time h	100 ppm Cl^−^				400 ppm Cl^−^				700 ppm Cl			
		E_cor_ V vs. SCE	i_cor_ μA cm^−2^	β_a_ mV	|β_c_| mV	E_cor_ V vs. SCE	i_cor_ μA cm^−2^	β_a_ mV	|β_c_| mV	E_cor_ V vs. SCE	i_cor_ μA cm^−2^	β_a_ mV	|β_c_| mV
CW617N	1	−0.111	0.69	33	167	−0.142	0.79	23	150	−0.158	1.08	23	151
	24	−0.122	0.70	70	237	−0.126	0.66	53	257	−0.141	0.88	56	256
CW602N	1	−0.095	0.74	45	187	−0.132	1.12	25	153	−0.132	1.10	20	150
	24	−0.105	0.62	55	256	−0.125	0.82	49	259	−0.124	0.85	43	211
CW724R	1	−0.109	0.65	32	166	−0.128	0.82	19	130	−0.148	0.80	21	147
	24	−0.101	0.59	58	269	−0.132	0.60	47	189	−0.140	0.80	24	151

**Table 3:** Fracture strains (ɛ_f_%) and SCC susceptibility index R (R = ε_fs_%/ε_fa_%) evaluated for the different brasses in SDW solutions containing 100, 400, and 700 ppm chlorides.

Environment (25 °C)	CW617N		CW602N		CW724R	
	ɛ_f_%	R	ɛ_f_%	R	ɛ_f_%	R
Air	17.2		46.9		45.6	
SDW (100 ppm Cl^−^)	4.6	0.27	30.4	0.65	39.9	0.88
SDW (400 ppm Cl^−^)	6.4	0.37	30.5	0.65	40.3	0.88
SDW (700 ppm Cl^−^)	5.6	0.32	28.3	0.60	37.3	0.82

## Data Availability

The data presented in this study are available on request from the corresponding author.
